# GreenGate - A Novel, Versatile, and Efficient Cloning System for Plant Transgenesis

**DOI:** 10.1371/journal.pone.0083043

**Published:** 2013-12-20

**Authors:** Athanasios Lampropoulos, Zoran Sutikovic, Christian Wenzl, Ira Maegele, Jan U. Lohmann, Joachim Forner

**Affiliations:** Centre for Organismal Studies, Heidelberg University, Heidelberg, Baden-Württemberg, Germany; Belgian Nuclear Research Centre SCK/CEN, Belgium

## Abstract

Building expression constructs for transgenesis is one of the fundamental day-to-day tasks in modern biology. Traditionally it is based on a multitude of type II restriction endonucleases and T4 DNA ligase. Especially in case of long inserts and applications requiring high-throughput, this approach is limited by the number of available unique restriction sites and the need for designing individual cloning strategies for each project. Several alternative cloning systems have been developed in recent years to overcome these issues, including the type IIS enzyme based Golden Gate technique. Here we introduce our GreenGate system for rapidly assembling plant transformation constructs, which is based on the Golden Gate method. GreenGate cloning is simple and efficient since it uses only one type IIS restriction endonuclease, depends on only six types of insert modules (plant promoter, N-terminal tag, coding sequence, C-terminal tag, plant terminator and plant resistance cassette), but at the same time allows assembling several expression cassettes in one binary destination vector from a collection of pre-cloned building blocks. The system is cheap and reliable and when combined with a library of modules considerably speeds up cloning and transgene stacking for plant transformation.

## Introduction

Ever since the first construction of a recombinant plasmid [1], [2], genetic engineering and molecular cloning mostly rely on the use of type II restriction endonucleases and DNA ligases. The DNA fragments to be combined are first excised from their precursor molecules via the endonucleases and then in a separate reaction re-assembled by the ligase, usually after spontaneous annealing of complementary single-stranded overhangs created during the endonuclease cut. While this approach is generally successful, there are certain limitations, especially when it comes to the assembly of complex plasmids from multiple elements, since with increasing numbers of DNA fragments the ligation reaction becomes less and less efficient. Thus, in case more than four DNA elements have to be assembled, success-rates drop significantly and additional rounds of cloning may be necessary. However, since most recognition sites used in a digestion-ligation cycle remain in the construct, the corresponding enzymes cannot be used for adding further DNA fragments in subsequent steps. Furthermore, many of these recognition sites occur fairly frequently in a given piece of DNA, making the assembly of long constructs even more difficult because of the lack of unique restriction targets. To overcome these limitations, researchers had to devise complicated cloning strategies to assemble plasmids, which involved many steps and required a large number of diverse restriction enzymes. With the advent of high throughput approaches and widespread use of transgenic models to test gene function *in vivo*, classical restriction based cloning rapidly became a major limitation and alternative technologies began to emerge.

One of the first ligation independent cloning methods was the univector plasmid fusion system, which is based on Cre/*loxP*-mediated recombination. Here, a gene of interest was cloned in the so-called pUNI vector and then transferred into a pHOST vector providing the regulatory sequences for expression [3], which allowed standardized and quick shuffling between a collection of entry and destination vectors. However, the 34 basepairs (bp) of the *loxP* sites lead to long cloning scars and the number of elements that can be combined is limited to two.

Another very popular recombination based cloning method is Invitrogen’s Gateway® technology [4], which exploits the lambda phage’s integration and excision mechanism. Briefly, the sequences to be joined need to be flanked by mutually exclusive variants of the attachment sites B and P or L and R. Two different enzyme mixes catalyze either the recombination between B and P sites (creating L and R sites) or between L and R sites (creating B and P sites). Often coding sequences are cloned into so-called entry vectors flanked by *attL1* and *attL2* sites and then transferred into destination vectors. These carry the regulatory sequences for expression and *attR1* and *attR2* sites between which the reading frame is inserted. After the recombination reaction, the insert is flanked by *attB1* and *attB2* sites of 25 bp each. A further development of this technique [5] – MultiSite Gateway® – allows the combination of up to five different fragments with more variants of the attachment sites. Gateway® cloning has been extensively used in many experimental systems, including plants, and large collections of Gateway® compatible vectors are available [6]. Despite this success, Gateway® cloning suffers from three main disadvantages: Firstly, the recombination sites leave 25 bp of unwanted junk sequence - so-called scars - and their inverted repeat sequence poses a problem for expression, sequencing, and RNA probe generation. Secondly, even in the Multisite flavour, the number of fragments that can be combined is limited and the reaction is fairly inefficient. And thirdly, the enzyme mixes required are rather expensive posing a limitation to many labs.

Another elegant way to avoid restriction enzymes and recombinases is ligation independent cloning (LIC) [7], [8]. This method makes use of the dual function of T4 DNA polymerase, as both 3′-5′ exonuclease and 5′-3′ DNA polymerase. The latter activity predominates in the presence of dNTPs. If only one kind of dNTP is added, the enzyme will digest the 3′-5′ strands of both ends of the DNA fragment until it reaches the first nucleotide for which dNTP is available. Thus single strand overhangs of defined length and sequence are created on both sides, usually 10–15 bp in length for cloning purposes. When fragments with compatible ends are mixed, they spontaneously anneal. Since mostly PCR products are used for LIC cloning, issues with sequence errors due to PCR or primers remain and in case modules are subcloned prior to the LIC reaction, suitable restriction enzymes are necessary to linearize the plasmids. Another issue are the long cloning scars dictated by 10–15 bp overhangs and severe limitations in combining multiple fragments at once.

A major breakthrough in cloning technology was the recent development of the Golden Gate method [9]. It allows fast and efficient assembly of a large number of building blocks requiring only two or three relatively cheap enzymes. The key components of this system are type IIS restriction endonucleases. These enzymes have specific recognition sites but cut arbitrary sequences at a defined distance from the recognition site. This offers several main advantages for cloning: One single enzyme is sufficient to release fragments from diverse preassembled modules and the recognition sites can be placed such that after ligation they cannot be cut again. This enables a one-way reaction of cutting and ligation in one tube, minimizing the experimental steps during the cloning procedure and increasing the efficiency of ligation even for large numbers of fragments. Since the overhangs created by these enzymes can be chosen freely because they are outside of the recognition site, the number of different fragments that can be combined in one reaction is very high. The overhangs generated by these enzymes are small, usually 4 bp, leaving only very short scars. Building blocks can be sub-cloned and sequenced prior to assembly, allowing labs to set up a library of ready-to-use components without the need to verify the final construct by sequencing.

Based on these Golden Gate mechanisms, we designed a simple and versatile cloning system for the generation of plant transformation vectors, which we named GreenGate. The GreenGate system allows rapid and efficient assembly of six modules typically representing promoter, N-terminal tag, coding sequence (CDS) (i.e. the gene of interest), C-terminal tag, plant terminator and plant resistance cassette into a T-DNA transformation plasmid. Furthermore, it offers the option to stack several of these expression cassettes onto a single T-DNA in subsequent rounds, dramatically reducing the time required to generate multi-construct transgenic plants.

## Results

### Golden Gate Principle

**Figure 1:**
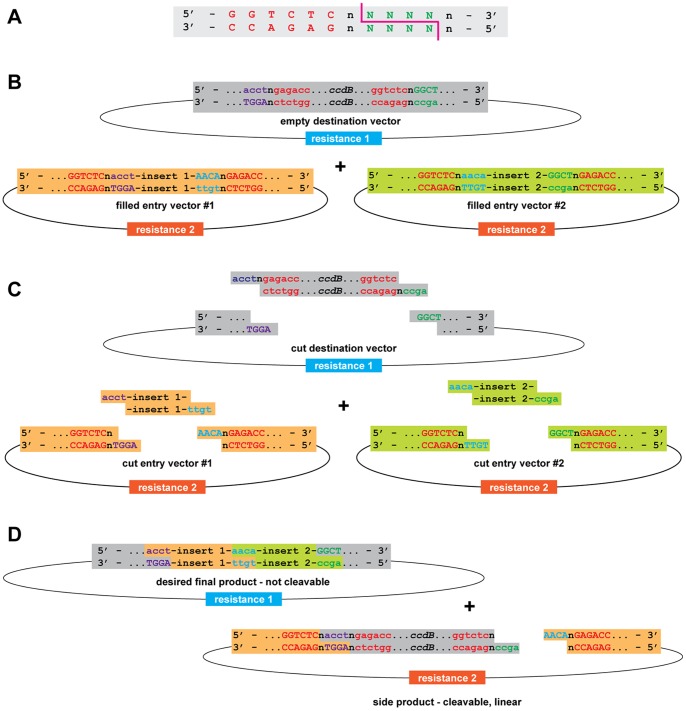
The Golden Gate principle. A) Type IIS restriction endonucleases, such as *Bsa*I, have a distinct, non-palindromic recognition site (red) and asymmetrically cut at a precisely defined distance regardless of the local sequence (green). *Bsa*I for instance creates a four base 5′-overhang starting from the second nucleotide downstream of the recognition site. B) A Golden Gate style cloning system requires two types of components, a destination vector and entry vectors containing the modules to be assembled. Each vector carries two recognition sites for the type IIS endonuclease (red) flanking the counter-selective marker on the destination vector and the modules on the entry vectors, respectively. Destination and entry vectors confer different markers for bacterial selection. The sequences in purple, blue and green represent the cutting sites. C) The orientation and position of the recognition sites is such that after digestion they remain with the backbone of the entry vectors, but are excised from the destination vector along with the counter-selectable marker (*ccdB*). D) The single stranded overhangs generated by the endonuclease can anneal to complementary sequences and be covalently linked by T4 DNA ligase. During the Golden Gate reaction in the presence of endonuclease and ligase the desired final product, but also the original vectors or a plethora of side-products (one of them shown at the bottom) can be created. However, only the desired final product is resistant to further endonucleolytic cleavage, whereas all other molecules will be cut again and again and thus will disappear from the reaction over time.

The Golden Gate cloning method allows the rapid and efficient assembly of constructs from pre-cloned building blocks in a single-tube reaction. The method is based on the use of type IIS restriction endonucleases to release DNA fragments from entry vectors and guide them to their specific position in the target plasmid. Type IIS enzymes bind to a defined recognition site, but cut the DNA strand at a fixed distance outside of the recognition motif regardless of the local sequence. This feature can be exploited for a cloning system in which several components are arranged into a single, pre-defined construct by simple incubation with a suitable destination vector, restriction endonuclease and ligase (Fig. 1).

Such a cloning system requires all inserts to be flanked by type IIS recognition sites in an orientation that fragments do not carry the sites after type IIS mediated release. This can be achieved either by cloning the fragments into entry vectors that harbour type IIS sites, or by incorporating them by PCR. Specific overhang sequences for each class of insert not only define the orientation of each fragment, but also the order in which they will be assembled in the final construct: Dependent on the intended position in the construct, each module is flanked by a different overhang at 5′- and 3′-end, while the overhangs of adjacent fragments are complementary. Thus, modules will only anneal to each other and the destination vector in the desired orientation and order, upon which ligase activity will complete the assembly. Importantly, no restriction enzyme recognition sites will be present in the final product after ligation, whereas re-ligated entry vectors retain restriction sites and thus can be cut over and over again. For GreenGate we designed the overhangs to be non-palindromic to avoid tandem ligation of inverted fragments and to differ by at least two out of the four bases to ensure specificity of annealing [10]. In contrast to the entry plasmids, the destination vector carries recognition sites in an orientation such that they are removed from the backbone after type IIS digest and that overhangs compatible to those of the outermost insert modules are exposed. In addition, the destination vector encodes an antibiotic resistance different from the one used for the entry vectors, as well as a negative selection marker on the insert. Thus, a reaction including entry vectors, destination vector, type IIS restriction enzyme and ligase will only produce a single product that is able to support bacterial growth after transformation in the presence of positive and negative selection specific to the destination vector. A limitation of Golden Gate cloning is that DNA fragments used as substrates for the reaction must be devoid of recognition sites for the type IIS restriction endonuclease used. This can be overcome by choosing an enzyme that rarely cuts the genome of the targeted model species and/or PCR based mutation of recognition sites prior to cloning.

In essence, Golden Gate cloning is suitable for assembling arrays of any kind of DNA fragments, including PCR products, but it is especially useful when creating multiple permutations of a finite number of building blocks. Constructing expression cassettes for transgenesis is a prime example for such a case, since a limited number of modules (promoters, coding sequences, tags, vector backbones etc.) need to be assembled in an invariant order. Leveraging combinatorial power and re-use of validated building blocks, Golden Gate cloning allows rapid and cost-efficient generation of complex constructs, which is easily scalable to meet high-throughput demands. To make these advantages accessible for the plant science community, we adapted the Golden Gate methodology and created GreenGate, a system for flexible and efficient assembly of plant transformation vectors.

### General GreenGate Design Principles

When designing the GreenGate system we aimed at creating a simple and efficient toolbox, which would allow the assembly of routine plant expression constructs, while at the same time facilitate the creation of more complex T-DNAs. Another important aim was to provide full flexibility in the choice of selectable markers for plant transformation, since this has proven to be one of the major bottlenecks in transgenic analyses.

The first important component for a Golden Gate cloning system is the type IIS restriction endonuclease, since it dictates the recognition site sequence. For GreenGate we chose the well characterized *Bsa*I (*Eco*31I), which recognizes a GC-rich six bp sequence (GGTCTCN^∧^NNNN) underrepresented in the genome of *Arabidopsis thaliana* (*A. thaliana*), and is commercially available through a number of suppliers. Consequently, DNA fragments containing a *Bsa*I site need to be modified by mutagenic PCR before cloning into the entry vector.

**Figure 2:**
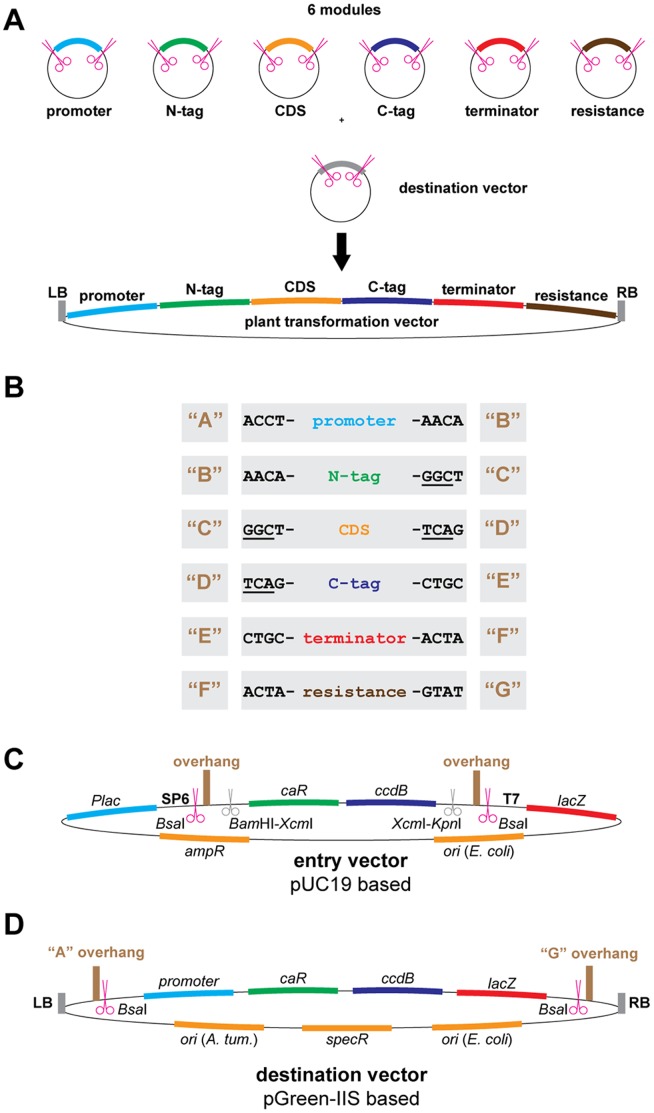
GreenGate vector design and layout. A) The GreenGate cloning system uses six different types of pUC19 based entry vectors into which the individual elements are inserted and a pGreen-IIS based destination vector. Magenta scissors represent *Bsa*I recognition sites. In each GreenGate reaction, six modules are ligated between the left border (LB) and the right border (RB) sequences of the destination vector yielding a ready-to-use plant transformation vector with expression unit and resistance cassette. These six modules encompass a plant promoter, an N-terminal tag, a coding sequence (i.e. the gene of interest), a C-terminal tag, a plant terminator and a plant resistance cassette for selection of transgenic plants. The modules can only be ligated in the pre-defined order. B) The orderly assembly is enabled by a set of seven different overhangs. Each module is flanked at its 5′-end by the same overhang as the 3′-end of its preceding neighbor. The individual overhangs all differ from each other by at least two out of the four nucleotides. The underlined nucleotides define coding triplets to which all other coding elements have to be in frame. C) Empty entry vector. The multiple cloning site of pUC19 has been replaced by two *Bsa*I recognition sites (magenta scissors), the respective overhangs for each module type and a counter-selectable *ccdB* gene. DNA fragments can be cloned via the specific overhangs, via the *Bam*HI and *Kpn*I sites or via A-overhangs after *Xcm*I digestion. *Plac* = *lac* promoter, SP6 = SP6 promoter, *caR* = *chloramphenicol acetyltransferase* gene, T7 = T7 promoter, *lacZ* = *lacZα* coding sequence, *ampR* = *beta-lactamase* gene, *ori* = origin of replication. D) Empty destination vector. A counter-selectable *ccdB*-cassette has been inserted between the LB and RB sequences of pGreen-IIS, flanked by *Bsa*I sites, with overhangs A and G. *promoter* = bacterial promoter. The pSa origin of replication (*ori A. tum.*) requires the presence of the helper plasmid pSOUP in agrobacteria.

The second important choice for GreenGate was the number of different modules that can be assembled into a single construct. Since the final plasmid can only be ligated when DNA fragments representing all modules are present in the reaction, dummy sequences need to be introduced if certain modules, e.g. tags, are not needed in a given construct. Thus, we wanted to limit the number of modules for rarely used functions to reduce junk sequences in our expression cassettes. Having a system in mind that would support day-to-day cloning, the number of modules should reflect the essential molecular functions of a gene. Consequently, in the GreenGate system six modules represent the plant promoter (1), an N-terminal tag (2), the coding sequence of the gene of interest (3), a C-terminal tag (4), the plant terminator (5) and the plant resistance cassette (6) (Fig. 2). However, with the exception of the resistance cassette, the modules could also take on other functions, if desired. In those cases where six modules are insufficient to assemble a construct, multiple functions can be bundled in a single module, e.g. by fusion PCR or Golden Gate style fragment joining before cloning into the corresponding entry vector. In addition, we incorporated the option to stack multiple expression cassettes onto a single T-DNA, allowing the generation of highly complex plasmids with a simple workflow that relies exclusively on *Bsa*I mediated fragment release.

### GreenGate Entry Vectors

We first established the six basal entry vectors into which DNA fragments need to be cloned to create the GreenGate modules (Fig. 2, A to C). As basis for the entry vectors, we chose pUC19, because it is a small high-copy plasmid that allows for blue-and-white selection [11] which is maintained in our design. pUC19 has been successfully used in molecular cloning for decades and confers resistance to ampicillin, which is compatible with the most commonly used *Escherichia coli* (*E. coli*) strains. To create the entry vectors, we replaced the multiple cloning site (MCS) by two *Bsa*I recognition sites and the respective module-specific overhangs, flanking a *chloramphenicol acetyltransferase* (*caR*)*-ccdB* cassette. This cassette allows counter-selection against the original vector in *ccdB* sensitive *E. coli* strains during the initial cloning of the DNA fragment of interest [12]. In addition, blue-and-white selection is possible, since the Shine-Dalgarno sequence and the start codon for *lacZα* are removed along with the *ccdB* cassette if the GreenGate reaction is successful. To create ready-to-use GreenGate modules, DNA fragments can be inserted into our entry vectors in three ways: 1) Addition of *Bsa*I sites and respective overhangs by PCR and cloning using these sites. 2) Fragments can be cloned via *Bam*HI and *Kpn*I sites present in the vectors. 3) A-tailed DNA fragments can be ligated after *Xcm*I-digest of the plasmid. Once the desired piece of DNA has been cloned into any of the entry vectors, it can be sequenced from both ends using standard T7 and SP6 primers. These promoters also allow for direct RNA probe generation using the corresponding polymerases. Since our vectors do not contain inverted repeat structures, sequencing as well as probe generation can be carried out with high efficiency. The entry vectors serve as a universal hub for the DNA fragments assigned to the diverse functions in the final construct, such as promoter, tag, or coding sequence. This identity is encoded in the specific overhangs, which are exposed after *Bsa*I digest. We designed the overhangs of the individual modules according to the following criteria: 1) All overhangs are non-palindromic to prevent inverted tandem ligation of the same insert. 2) At least two out of four nucleotides differ between overhangs to avoid mis-ligation. 3) We optimized the sequence of the overhangs for functionality, if applicable: The B overhang between promoter and N-tag was designed to be the plant Kozak consensus sequence (AACA) [13]; C and D overhangs between N-tag and CDS and CDS and C-tag, respectively, encode for glycine or serine codons, commonly used in linker sequences. Furthermore, we designed all N- and C-terminal tags to carry start and stop codons, respectively, and consequently have removed stop codons from all CDS modules to make them fully compatible with our tags. The start codon of the CDS module, however, needs to be retained, since not all B-modules carry an ATG codon. For cases where no tags are desired, we designed dummy inserts for modules B and D (B-dummy and D-dummy), which can replace the tags by short sequences. Since in our design the CDS (module C) is devoid of a stop codon, the D-dummy carries a stop codon to provide this function.

### Destination Vectors

For the destination vectors, we chose the plant transformation vector pGreen-IIS as backbone [14] [15]. This vector worked well in our hands both for *A. thaliana* floral dip transformation and *Nicotiana benthamiana (N. benthamiana*) leaf infiltration. A major advantage of pGreen-IIS is its reduced backbone sequence, which only contains the spectinomycin resistance and origins of replication for *E. coli* and *Agrobacterium tumefaciens* (*A. tumefaciens*). The replicase required for growth in *A. tumefaciens* needs to be provided in *trans*, e.g. on the pSOUP plasmid [14]. The small backbone allows for a larger T-DNA which will be transferred into the plant genome. To create a GreenGate compatible destination vector, we replaced the sequence between the left border (LB) and the right border (RB) of the T-DNA by a *caR*-*ccdB*-*lacZα* cassette flanked by two *Bsa*I recognition sites in opposite orientation and A and G overhangs (Fig. 2D). We made two versions of the destination vector, one where the plant resistance cassette is located at the RB of the T-DNA (pGGZ001) and one where it is located at the LB (pGGZ003). With such a design both *ccdB* counterselection in a *ccdB* sensitive strain and blue-and-white screening are possible to discriminate against the original vector after the GreenGate reaction, which reduces the background of undesired colonies. All available GreenGate vectors are listed in Table 1.

**Table 1:** Available GreenGate vectors and modules.

	Name	Type	Overhangs^a^	Bacterialresistance	Addgene ID
**Empty entry** **vectors (** ***ccdB*** **^+^)**	pGGA000	Plant promoter	A–B	Ampicillin	48856
	pGGB000	N-tag	B–C	Ampicillin	48857
	pGGC000	CDS	C–D	Ampicillin	48858
	pGGD000	C-tag	D–E	Ampicillin	48859
	pGGE000	Plant terminator	E–F	Ampicillin	48860
	pGGF000	Plant resistance cassette	F–G	Ampicillin	48861
	pGGH000	N-tag+CDS+C-tag+terminator	B–F	Ampicillin	48862
	pGGI000	N-tag+CDS+C-tag	B–E	Ampicillin	48863
**Empty intermediate** **vectors for two** **expression cassettes** **on one T-DNA (** ***ccdB*** **^+^)**	pGGM000	Assembly of expression cassette #1(without plant resistance)	A–H	Kanamycin	48864
	pGGN000	Assembly of expression cassette #2(with plant resistance)	H–G	Kanamycin	48865
**Empty destination** **vectors (** ***ccdB*** **^+^)**	pGGY001	Plant resistance at RB^b^	A–G	Gentamicin	48866
	pGGY003	Plant resistance at LB^c^	A–G	Gentamicin	48867
	pGGZ001	Plant resistance at RB	A–G	Spectinomycin	48868
	pGGZ003	Plant resistance at LB	A–G	Spectinomycin	48869
**Plant promoters**	pGGA002	*AP3* (*APETALA3*; internal *Bsa*Isite removed) promoter	A–B	Ampicillin	48813
	pGGA003	*WUS* (*WUSCHEL*; 4.4 kb) promoter	A–B	Ampicillin	48814
	pGGA004	*35S (Cauliflower mosaic virus 35S*;internal *Bsa*I site removed) promoter	A–B	Ampicillin	48815
	pGGA006	*UBQ10* (*UBIQUITIN10*) promoter	A–B	Ampicillin	48816
	pGGA008	*ALCA* (*A. nidulans alc* regulon) promoter	A–B	Ampicillin	48817
	pGGA012	*RPS5A* (*RIBOSOMAL PROTEIN 5A*) promoter	A–B	Ampicillin	48818
**N-tags**	pGGB001	*mCherry-linker*	B–C	Ampicillin	48819
	pGGB002	*Ω* (*Omega- element)*	B–C	Ampicillin	48820
	pGGB003	*B-dummy* (default random sequence if nospecific N-tag is desired)	B–C	Ampicillin	48821
	pGGB005	*SV40 NLS (SIMIAN VIRUS 40 NUCLEAR* *LOCALIZATION SIGNAL)*	B–C	Ampicillin	48822
	pGGB006	*ER signal sequence*	B–C	Ampicillin	48823
**Coding sequences**	pGGC001	*WUS* (internal *Bsa*I site removed)	C–D	Ampicillin	48824
	pGGC011	*ALCR (A. nidulans alc regulon* *transcriptional regulator)*	C–D	Ampicillin	48825
	pGGC012	*GFP-NLS (GREEN FLUORESCENT* *PROTEIN-NUCLEAR LOCALIZATION SIGNAL)*	C–D	Ampicillin	48826
	pGGC014	*GFP (A206K)*	C–D	Ampicillin	48827
	pGGC015	*mCherry*	C–D	Ampicillin	48828
	pGGC024	*BFP (BLUE FLUORESCENT PROTEIN)*	C–D	Ampicillin	48829
	pGGC025	3x *GFP*	C–D	Ampicillin	48830
	pGGC026	3x *mCherry*	C–D	Ampicillin	48831
	pGGC051	*GUS (E. coli ß-GLUCURONIDASE)*	C–D	Ampicillin	48832
**C-tags**	pGGD001	*linker-GFP*	D–E	Ampicillin	48833
	pGGD002	*D-dummy* (default random sequence withstop codon if no specific C-tag is desired)	D–E	Ampicillin	48834
	pGGD003	*Linker-mCherry*	D–E	Ampicillin	48835
	pGGD005	*Linker-BFP*	D–E	Ampicillin	48853
	pGGD006	*SV40 NLS*	D–E	Ampicillin	48836
	pGGD007	*linker-NLS*	D–E	Ampicillin	48837
	pGGD008	*ER retrieval signal* (HDEL)	D–E	Ampicillin	48838
**Plant terminators**	pGGE001	*RBCS* terminator (from pea)	E–F	Ampicillin	48839
	pGGE002	*WUS* terminator	E–F	Ampicillin	48840
	pGGE003	*At1g04880* terminator	E–F	Ampicillin	48854
	pGGE005	*At1g76110* terminator	E–F	Ampicillin	48855
	pGGE009	*UBQ10* terminator	E–F	Ampicillin	48841
**Plant resistance** **cassettes**	pGGF001	*p* d *MAS* e *:BastaR* f *:t* g *MAS*	F–G	Ampicillin	48842
	pGGF002	*p35S* (internal *Bsa*I site removed*):BastaR:t35S*	F–G	Ampicillin	48843
	pGGF003	*pMAS:D-AlaR* h *:tMAS*	F–G	Ampicillin	48844
	pGGF004	*p35S* (internal *Bsa*I site removed*):D-AlaR:t35S*	F–G	Ampicillin	48845
	pGGF005	*pUBQ10:HygrR* i *:tOCS* j	F–G	Ampicillin	48846
	pGGF007	*pNOS* k *:KanR* l *:tNOS*	F–G	Ampicillin	48847
	pGGF008	*pNOS:BastaR* (chi sequence removed):*tNOS*	F–G	Ampicillin	48848
	pGGF012	*pMAS:SulfR* m *:t35S*	F–G	Ampicillin	48849
**Adapters**					
	pGGG001	*FH-adapter*	F–H	Ampicillin	48850
	pGGG002	*HA-adapter*	H–A	Ampicillin	48851

a four nucleotide 5′-overhangs exposed at the cutting sites after *Bsa*I digestion, see Fig. 2 for exact nucleotide sequence.

b T-DNA right border.

c T-DNA left border.

d promoter.

e *mannopine synthase.*

f *phosphinotricine acetyltransferase.*

g terminator.

h *D-amino acid oxidase.*

i *hygromycinB phosphotransferase.*

j *octopine synthase.*

k *nopaline synthase.*

l *neomycin phosphotransferase II.*

m *dihydropteroate synthase.*

### Plant Resistance Cassettes

One of the main issues we wanted to address with the GreenGate system was to facilitate transgene stacking. Since most plant transformation vectors confer resistance to either Basta™ or kanamycin, selecting transgenic events in secondary or tertiary transformations (e.g. into lines from the Salk [16] or SAIL [17] T-DNA insertion mutant collections) can be challenging. To overcome these limitations we designed a collection of different plant resistance cassettes as GreenGate modules, which can now be included into the assembly of any transformation vector. We chose five selectable markers that work well in our hands, namely *phosphinotricine acetyltransferase* (*BastaR*) conferring resistance against the herbicide Basta™, *neomycin phosphotransferase II* (*kanR*) against kanamycin, *hygromycinB phosphotransferase* (*hygR*) to hygromycin, *dihydropteroate synthase* (*SulfR*) allowing growth on sulfadiazine [18], [19] and *D-amino acid oxidase* (*D-alaR*) detoxifying D-alanine [20]. Since multiple copies of identical sequences in transgenes can trigger gene silencing [21], we designed the cassettes in a way to maximize regulatory sequence divergence for these markers. In addition to the *nopaline synthase* (*NOS*) promoter and terminator, we used the *mannopine synthase* (*MAS*) and *cauliflower mosaic virus 35S* (*35S*) promoters and terminators, the *UBIQUITIN10* (*UBQ10*) promoter and the *octopine synthase* (*OCS*) terminator in different combinations. We also removed the *chi* sequence [22] that potentially enhances recombination in bacteria from the *phosphinotricine acetyltransferase* coding sequence in one of the cassettes. An overview over available resistance cassettes is given in Table 1.

To test if all eight resistance cassettes are fully functional when selecting T1 plants, we created a construct harboring the promoter (*p*) *of APETALA3* (*AP3*) fused to *mCherry,* which was connected to the *WUSCHEL* (*WUS*) cDNA by a linker coding for 34 amino acids followed by the same linker and *GREEN FLUORESCENT PROTEIN* (*GFP*). As transcriptional terminator (*t*) we used 650 bp of the 3′-downstream region of the *RIBULOSE-1,5-BISPHOSPHATE CARBOXYLASE OXYGENASE SMALL CHAIN* (*RBCS*) gene. This *pAP3:mCherry-linker-WUS-linker-GFP:tRBCS* expression cassette was combined with all eight resistance modules. We recovered resistant seedlings for all of the modules and nearly all transgenic plants displayed the expected phenotype (see below). The availability of a wide range of selectable markers for plant transgenesis will support simple generation of double and triple transgenic plants.

### Proof of Principle

After establishing all basic components for GreenGate, we tested them in a number of applications. To this end, we generated functional modules for all classes and assembled them into plant transformation constructs via GreenGate reactions. The pTL003 plasmid coded for an *mCherry-linker-WUSCHEL-linker-GFP* fusion driven by the flower specific *AP3* promoter and flanked on the 3′-end by the *RBCS* terminator [23]. We chose the resistance to the popular herbicide Basta™ as plant selectable marker, which was flanked by *MAS* regulatory sequences. For pTL004 we used the same coding modules combined with the *WUS* promoter and terminator [24], as well as the *pUBQ10:hygR:tOCS* resistance module. pTL005 was assembled using the *35S* promoter, *mCherry-linker* [25], the *WUS* ORF, the D-dummy module and the *RBCS* terminator combined with the *pMAS:D-alaR:tMAS* resistance module. We obtained more than a hundred colonies after transformation into chemically competent *E. coli* for each GreenGate reaction. We randomly picked eight colonies for each construct and checked for the presence of the desired insert by colony PCR. Since all colonies tested were positive, we isolated plasmid DNA from four clones each and analyzed it by several test digestions. In all but one instance digestion patterns matched the expected sequence, which was finally confirmed by Sanger sequencing of a single clone for each construct.

**Figure 3:**
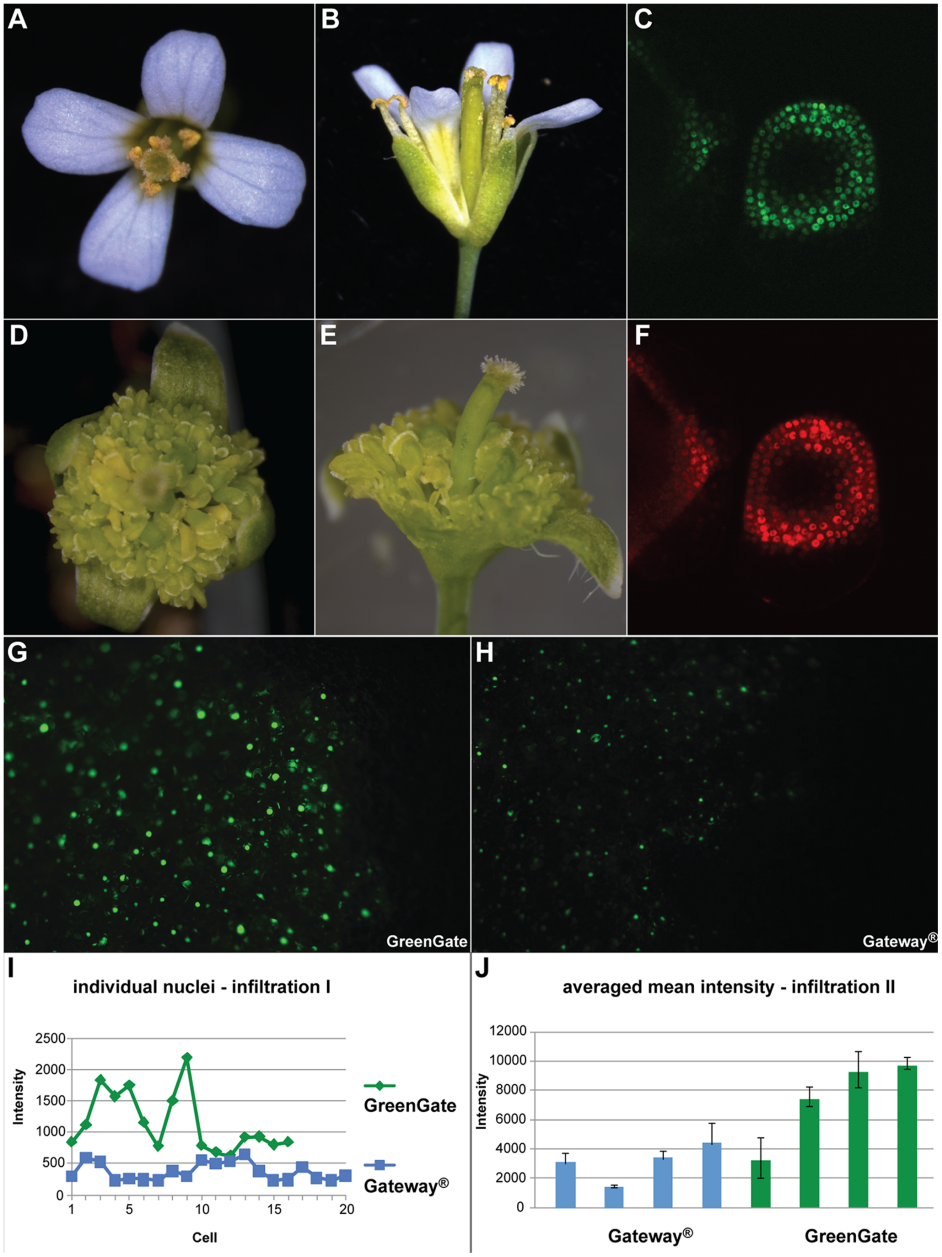
Proof of concept. pTL003 (a *pAP3:mCherry-linker-WUS-linker-GFP:tRBCS*; *pMAS:BastaR:tMAS* construct) served as positive control for the GreenGate concept. When compared to wild-type (A and B), 61 of the 62 T1 transformants displayed a severe *pAP3:WUS*-flower phenotype (D and E), where instead of four petals and six stamen a large number of carpelloid floral organs is formed. Confocal microscopy of inflorescence apices revealed GFP (C) and mCherry (F) expression in whorls two and three of floral buds. *AP3* = *APETALA3*, *WUS* = *WUSCHEL*, *RBCS* = *RuBisCO small subunit*. Two identical constructs were created with either GreenGate or a 2-component Gateway®-based cloning method. pTL013 (*pUBQ10:B-dummy-GFP-NLS-D-dummy:tRBCS*; *pMAS:BastaR:tMAS*) for GreenGate, pJF343 (*pUBQ10:attB1-GFP-NLS-attB2:tRBCS*; *pNOS:BastaR:tNOS*) in case of Gateway®. *UBQ10* = *UBIQUITIN10*, *NLS* = *nuclear localization signal*. Pictures of *Nicotiana benthamiana* leaves were taken three days after infiltration with pTL013 (G) and pJF343 (H); the signal from leaves infiltrated with the GreenGate derived construct is visibly brighter. Quantification of the fluorescence intensity of single nuclei from both approaches was done by confocal laser scanning (I) and epifluorescence microscopy (J) in two independent experiments. The signal from the GreenGate derived construct is stronger, and intensity ranges between both approaches hardly overlap.

All three GreenGate constructs were used for *A. thaliana* transformation. From 500 µL of T1 seeds sown out for each construct on selective medium, we recovered 62 transgenic seedlings for pTL003, eight for pTL004 and none for pTL005. 61 of the 62 pTL003 plants exhibited the strong *pAP3:WUS* phenotype (Fig. 3, D and E). Instead of 4 petals and 6 stamens, their flowers showed a large number of carpelloid organs and were mostly infertile [26]. As expected from the addition of the N-terminal *mCherry* and the C-terminal *GFP* tags, pTL003 positive plants also showed red and green fluorescence in the nuclei of cells in whorls 2 and 3 in floral buds (Fig. 3, C and F). We were able to recover a small number of seeds from two independent T1 plants and could confirm the *AP3:WUS* floral phenotype in T2. Transgenic plants recovered from the transformation with pTL004 did not show a phenotype, as expected, since here the endogenous *WUS* promoter drives a fluorescently labelled version of WUS. Confocal microscopy revealed that all plants showed mCherry and GFP activity in nuclei of the organizing centre, in line with the expected *WUS* expression pattern (not shown). The low number of T1 transgenics recovered in our experiment is consistent with our experience with transgenes containing the *WUS* promoter in other vector systems. Similarly, we were not surprised to be unable to recover transgenic plants after transformation with pTL005, since ubiquitous expression of *WUS* is lethal. However, when we infiltrated *N. benthamiana* leaves with agrobacteria carrying this construct, we detected mCherry activity in a large number of nuclei (not shown), showing that the expression cassette on the T-DNA is functional.

Furthermore, we created two test constructs to compare the expression strength between a GreenGate-derived plasmid (pTL013) and one generated by Gateway®-cloning (pJF343). Both constructs contain the same elements and use the *UBQ10* promoter to drive expression of nuclear localized GFP. Thus, the main difference between the two transcriptional units are the *attB1* and *attB2* sites flanking the coding sequence in the Gateway® assembled plasmids. When *N. benthamiana* leaves were infiltrated with these constructs, the fluorescence from the GreenGate version was visibly brighter when compared to the Gateway® derived clones (Fig. 3, G and H) and quantification of fluorescence intensities supported this impression (Fig. 3, I and J).

Since in many experiments N- and C-terminal tags are not necessary, we also compared the influence of our dummy sequences, the B-dummy (pGGB002), the Ω-element (pGGB003), a translational enhancer from *tobacco mosaic virus (U1)* [27], and the D-dummy (pGGD002), by using the *pAP3:WUS* phenotypes as a quantitative readout. In constructs analogous to pTL003, the N-terminal *mCherry-linker* tag was replaced by either the B-dummy (pZS165) or the Ω-element (pZS163). When scoring for the strength of the *pAP3:WUS* phenotype, we found 106 plants out of 134 ( = 79%) showing the strong phenotype for pZS165 and 54 out of 98 ( = 55%) for pZS163, suggesting that the inclusion of the Ω-element did not enhance protein expression in this scenario. When we exchanged the C-terminal *linker-GFP* tag for the D-dummy to create pZS164, nearly all T1 plants (78 out of 79) showed the strong phenotype as was the case for pTL003, which carried the doubly tagged form of WUS.

### Two Expression Cassettes on One T-DNA

To speed up transgene stacking we devised a system to place multiple expression cassettes on a single T-DNA. We aimed to achieve this using our pre-cloned modules without the need to set up a parallel system with different overhangs or recognition sites for a second type IIS enzyme.

**Figure 4:**
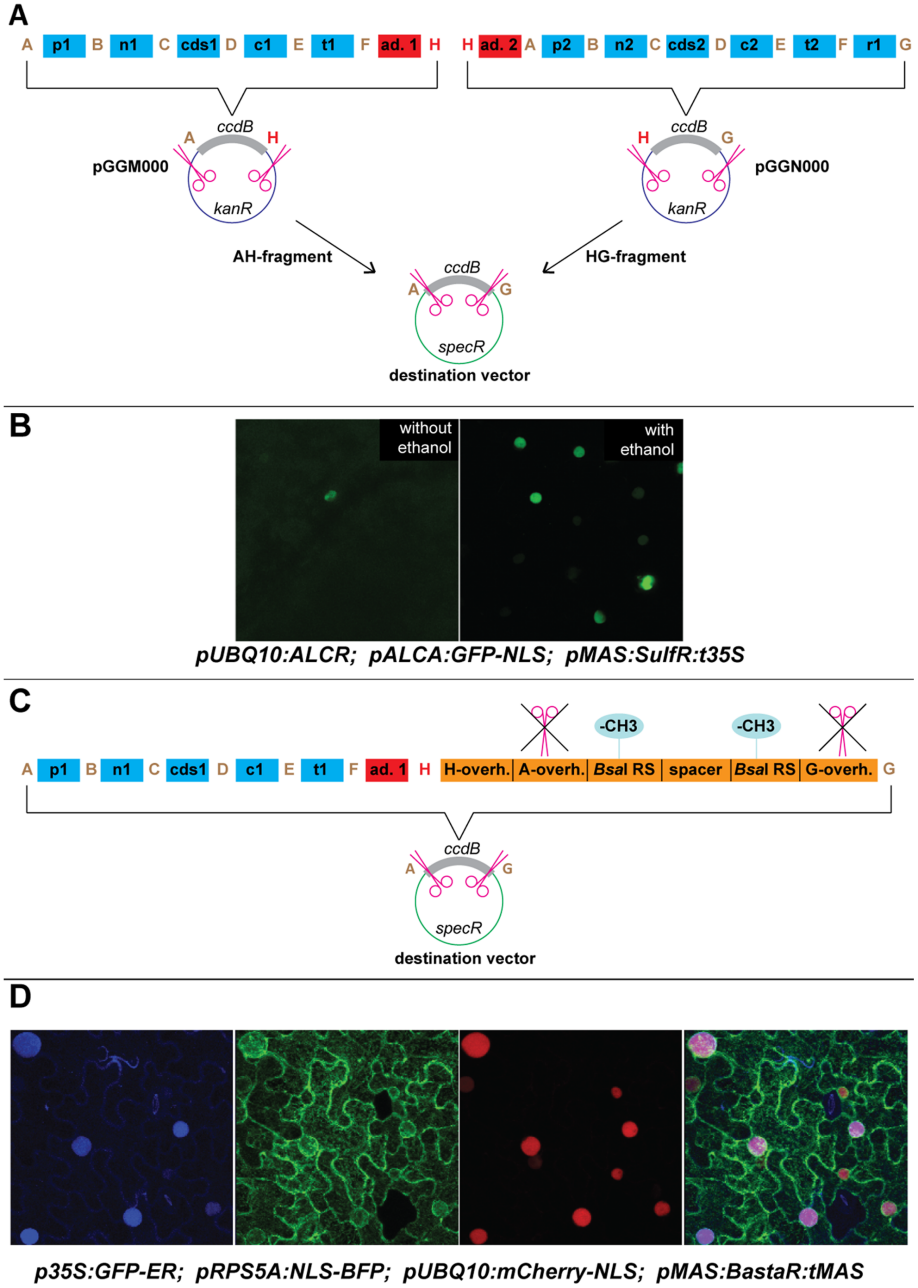
Multiple expression cassettes on a single T-DNA. A) The first strategy uses one additional overhang (“H” = TAGG), two adapter modules and two intermediate vectors. In a first step, two expression cassettes (“supermodules”) are assembled in parallel in two different intermediate vectors (pGGM000 and pGGN000). The *Bsa*I sites in the intermediate vectors are retained in the supermodule. In the second step, these two supermodules are then transferred into a destination vector via a normal GreenGate reaction. The overhang types are given in capital letters. p1/2 = promoter, n1/2 = N-terminal tag, cds1/2 = coding sequence, c1/2 = C-terminal tag, t1/2 = terminator, r1 = plant resistance, ad.1 = FH-adapter module, ad.2 = HA-adapter module. B) Fluorescence microscopy images show *Nicotiana benthamiana* leaves infiltrated with a construct harboring two expression cassettes on one T-DNA created via this method. The images were taken 72 hours after infiltration and 24 hours after ethanol induction (picture on the right). The first transcriptional unit drives constitutive expression of the ALCR transcription factor (*pUBQ10:B-dummy-ALCR-D-dummy:tRBCS*; *pMAS:sulfR:t35S*), the second one (*pALCA:Ω-element-GFP-NLS-D-dummy:tRBCS*) of nuclear localized GFP in presence of ethanol-bound ALCR protein. C) Only one additional element is required for the second strategy. Instead of a plant resistance cassette module, the FH-adapter module from strategy #1 and an oligo duplex (orange) with unpaired H and G overhangs are used in the GreenGate reaction. The oligo duplex contains internal *Bsa*I sites that would result in A and G overhangs after digestion. However, digestion is blocked by methylation of the cytosine residues in the *Bsa*I recognition sites, since *Bsa*I is sensitive to methylation. After transformation of the resulting construct into bacteria, the methylation is lost during replication because no *dcm* site is present. Thus, after re-isolation from bacteria, the plasmid, already containing one expression cassette, can function as an empty GreenGate destination vector, releasing A and G overhangs after digestion by *Bsa*I and removal of the *Bsa*I recognition sites from the vector backbone. This process can in principle be re-iterated infinitely. The construct is finalized by using a standard plant resistance module in the last step. D) *N. benthamiana* leaves infiltrated with a destination vector (pTL019) carrying three transcriptional units assembled by this method. The fluorescence signal from all three individual expression cassettes, i.e. nuclear localized BFP (left), ER-localized GFP (second from left) and nuclear localized mCherry (third from left), is visible in all transformed cells. Merge shown on the right.

In a first attempt we limited ourselves to two expression cassettes on one T-DNA plasmid (Fig. 4A). To this end, we created two intermediate vectors, pGGM000 and pGGN000, and two adapter molecules. The final construct is created in two steps: First, the two transcriptional units are assembled separately in two parallel reactions into the intermediate vectors. The first of these two supermodules consists of a plant promoter, an N-terminal tag, a coding sequence, a C-terminal tag and a plant terminator. Furthermore, a pre-cloned adapter module with F and H overhangs is added to the reaction. The intermediate vector has *Bsa*I recognition sites remaining in the vector backbone with matching A and H overhangs and confers resistance to kanamycin. The elements are combined in a GreenGate reaction but because two *Bsa*I sites remain in the final construct, an additional ligation reaction is performed before transformation. The second supermodule is built similarly, but the adapter molecule has H and A overhangs and at the 3′-end a plant resistance cassette is added. Thus, the resulting plasmid will have H and G overhangs. The two supermodules are then combined with the destination vector in a second step in a standard GreenGate reaction yielding the final construct.

As a proof of concept we made an ethanol-inducible *GFP-nuclear localization signal* (*NLS*) construct, using the *ALCA* promoter and the ALCR transcription factor from the *Aspergillus nidulans alc* regulon [28] and the *Ω-element*. First supermodules pGGM002 (*pALCA:Ω-element-GFP-NLS-D-dummy:tRBCS*; FH-adapter) and pGGN002 (HG-adapter; *pUBQ10:B-dummy-ALCR-D-dummy:tRBCS*; *pMAS:sulfR:t35S*) were created. These two were combined with the destination vector pGGZ001 to give rise to pTL016 (*pALCA:Ω-element-GFP-NLS-D-dummy:tRBCS*; FH-adapter; HG-adapter; *pUBQ10:B-dummy-ALCR-D-dummy:tRBCS*; *pMAS:sulfR:t35S*).

Cloning the two intermediate supermodules was not as efficient as the standard GreenGate reactions. We observed a reduced absolute number of transformants and among these in one case more than 50% had aberrant plasmid digestion patterns. Combining the two supermodules with the destination vector, however, was again extremely efficient.

*N. benthamiana* leaves from four plants were infiltrated with agrobacteria carrying this construct. 48 hours after infiltration half of the plants were watered with 1% ethanol to induce GFP expression, the other half served as control. GFP fluorescence was clearly visible 24 hours later in the nuclei of the induced leaves, but rarely in the control. One control plant was then watered with 1% ethanol and checked for fluorescence again 24 hours later. This time the nuclei were brightly green, showing that the double expression construct works as designed (Fig. 4B).

### More than Two Expression Cassettes on One T-DNA

To combine even more transcriptional units on one plasmid the way described for the double expression constructs, the system would have required a substantial number of additional overhangs and supermodule vectors, while at the same time limiting ourselves to a fixed maximum of expression cassettes. To avoid these complications and to retain full flexibility with regard to transgene complexity, we came up with a much simpler procedure, which required only a single additional element (Fig. 4C). This module is a synthetic DNA duplex with unpaired ends that allows ligation to H and G overhangs during a GreenGate reaction. Furthermore, it contains two internal *Bsa*I recognition sites, which cannot be cut by *Bsa*I due to overlapping cytosine methylation. Thus, the element will behave as an ordinary H to G module in the first GreenGate reaction and replace the resistance cassette. However, after isolation of the resulting plasmid from bacteria the methylation will be lost because no *dcm* sites are present in the oligo duplex. Consequently the two internal *Bsa*I recognition sites are now targets for *Bsa*I in a second round of GreenGate, giving rise to A and G overhangs, simulating an empty destination vector. Thus, in each subsequent GreenGate round either the uncleavable oligo duplex can be used to prepare the plasmid for the insertion of another expression cassette or the plasmid can be finalized by adding a plant resistance cassette to the reaction. In this approach, the transcriptional units are assembled serially, so it requires one additional step for each expression cassette to be added. The FH-adapter used along with the oligo duplex separates the individual expression units in the final construct and thus can be freely designed to act as a spacer of arbitrary length and sequence minimizing the risk of mutual interference of the expression behavior.

We tested this approach with a triple expression construct, namely pTL019 (*p35S:ER signal sequence-GFP-ER retrieval signal:tRBCS*; FH-adapter; *pRPS5A:SV40 NLS-E. coli biotin ligase-linker-BFP:TAt1g04880*; FH-adapter; *pUBQ10:B-dummy-mCherry-linker-NLS:tAt1g76110*; *pMAS:BastaR:tMAS* in pGGZ003). The expression cassettes were assembled in the given order using the serial strategy described above. Similar to our experience with using supermodules, cloning was not as efficient as for single unit plasmids. Again, we observed a severely reduced number of transformants, and many clones gave rise to an aberrant digestion pattern. In steps two and three we also observed a high percentage of the original destination vector, even after including an additional *Bsa*I digestion step after the GreenGate reaction. Once we had identified a correct clone, the final construct was used for infiltration of *N. benthamiana* leaves. Fluorescence was analyzed 64 hours after infiltration (Fig. 4D) and all three fluorescent proteins were clearly detectable in the subcellular compartments predicted by their primary sequence. Thus with these two approaches it is possible to create ready-to-use constructs containing multiple expression cassettes within 2–3 weeks.

## Discussion

We established GreenGate, a novel cloning system for plant transgenesis that supports easy, quick, modular and reliable assembly of plant expression cassettes directly in plant transformation vectors. Several independent and complex constructs have been successfully created and were functional in stable transformation of *A. thaliana* and transient essays in *N. benthamiana* leaves, respectively.

GreenGate is designed to match the requirements of routine and advanced cloning for plant transgenesis and, therefore, we adapted the Golden Gate [10] layout to encompass the six most frequently used elements in plant expression cassettes, namely plant promoters, N-terminal tags, coding sequences of the gene of interest, C-terminal tags, plant terminators and resistance cassettes for selection of transgenic plants. Furthermore, unlike in the MoClo [29] or GoldenBraid systems [30], where two or three different enzymes are used, GreenGate makes use of only one type IIS restriction endonuclease minimizing the likelihood of naturally occurring restriction sites in inserts, which would interfere with the reaction and thus need to be removed prior to cloning. Nevertheless, like the MoClo [29] or GoldenBraid systems [30], GreenGate allows stacking of several transcription units onto one construct and generation of libraries of re-usable higher-order supermodules. Since agrobacteria in our hands only tolerate pGreen-IIs based constructs of up to 20 kb, the number of possible expression cassettes on a single construct is, however, limited irrespective of the cloning system.

When comparing GreenGate to the most frequently used cloning systems for plant transgenesis so far, namely classical cloning and 2-component Gateway®, the main advantages are the full modularity, including the plant selectable marker, the fact that unwanted “scar” sequences are kept to a minimum and that entry clones can serve as templates for high quality sequencing and probe generation. While classical cloning allows sequencing and RNA probe generation, individual cloning strategies have to be worked out for every construct and the limited availability of unique restriction sites may severely reduce options for assembly of more complex transgenes. In contrast, 2-component Gateway®, is independent of restriction site limitations when it comes to cloning an insert into a given destination vector, but still promoter, terminator and resistance cassette need to be cloned traditionally during the design of this element, severely limiting the modularity of the system. In addition, long inverted-repeat recombination sites hamper sequencing and probe generation from entry clones and since they remain in the final construct also affect transgene function. This notion was supported by our finding that GreenGate assembled constructs not only lead to higher fluorescence intensities in transiently expressing leaf mesophyll cells of *N. benthamiana* (Fig. 3, G–J), but also produced a higher frequency of transgenic lines with strong *AP3:WUS* phenotypes in *A. thaliana*. Similar to Gateway®, ligation independent cloning leaves large scars in the constructs and either requires restriction enzyme sites to be removed when inserts are pre-cloned or to rely on PCR products without prior sequencing. Both systems in addition do not offer full modularity for assembly of constructs and thus require a large number of destination vectors to be designed and produced. In contrast, the MultiSite Gateway® cloning system allows five elements to be combined, but is rather expensive, in our hands rather inefficient, and still suffers from the cloning scars discussed above. GreenGate overcomes most of these limitations and thus should greatly facilitate the assembly of plant transformation constructs.

While being able to quickly assemble a large number of constructs expands our experimental scope, functional studies in plants often require two or more expression units to be tested together, for example regulator-reporter combinations, making plant transgenesis the next bottleneck. Co-transformation is a way to avoid time-consuming crossing of plants transformed with individual constructs, however, this requires the use of multiple plant resistance cassettes and co-transformation efficiency only lies in the range of 20–30% [31]. Combination of several transgenes in general can cause problems with gene silencing caused by multiple copies of identical sequences, for example when resistances are driven by the same promoter [21]. Thus, minimizing the number of resistance cassettes is an important aspect of experimental design. Therefore, we expanded our system to enable cloning of multiple transcriptional units on the same T-DNA from our established module library. The advantages in the downstream analysis of the transgenic plants in our opinion vastly outweighs the disadvantages, which lie in the substantially reduced efficiency of assembling supermodules or working with the methylated synthetic oligo duplex element. On the upside, once the supermodules are generated, they can be re-used just like ordinary modules at the same high efficiency.

After having routinely used GreenGate in the laboratory for more than eight months, we find it working well as everyday technique. However, especially when several people share components, care has to be taken to ensure a certain minimal standard for all elements. We have identified plasmid DNA quality and quantity to be an important determinant of GreenGate efficiency with a DNA concentration of at least 100 ng/µl after column preparation being a minimum requirement. Even more importantly, the activity of the restriction endonuclease is a limiting factor for GreenGate cloning. We found both *Bsa*I-HF from NewEngland Biolabs, as well as the isoschizomer FastDigest *Eco*31I from Fermentas to be highly sensitive to temperature fluctuations in a batch dependent manner. Thus, we highly recommend preparing small aliquots of all components, including the enzymes to avoid repeated freeze thaw cycles to ensure efficient and reliable cloning using GreenGate.

## Materials and Methods

### Construction of Empty Entry Plasmids

pUC19 [11] was chosen as basis for the entry constructs. In the first step, the *Bsa*I site in the *beta lactamase* (*ampR*) coding sequence was removed and converted into an *Xho*I site by two silent mutations via PCR. Apart from that no changes were made to the vector backbone. In the same step, the N-terminal part of the *lacZα* open reading frame around the multiple cloning site was replaced by *Hind*III and *Eco*RI sites flanked by SP6 and T7 promoter sequences. The *lacZα* Shine-Dalgarno sequence and translation start codon were deleted, the rest of the *lacZα* expression cassette was left unchanged. Next, the *Bsa*I recognition sites in opposite orientation with the respective overhangs for the six basal entry vectors were inserted, separated by *Bam*HI and *Kpn*I sites. In the third step, the *Xcm*I sites, the Shine-Dalgarno sequence for *lacZα* and *Xba*I and *Pst*I sites were added. The vectors were finalized integrating the open reading frames for *chloramphenicol acetyltransferase* (*caR*) and *ccdB*. The internal *Bsa*I site in *ccdB* was destroyed by a silent nucleotide exchange.

Vector elements were either generated via PCR amplification or via artificially synthesized oligo duplices; all constructs were verified by sequencing and test digestion. PCRs were done with Phusion High-Fidelity DNA Polymerase (Fisher Scientific - Germany GmbH, Schwerte, Germany). PCR products and intermediate plasmids were cut by conventional restriction endonucleases (obtained from Fisher Scientific - Germany GmbH, Schwerte, Germany) and ligated with T4 DNA ligase (Fisher Scientific - Germany GmbH, Schwerte, Germany). For complex PCR products, the single elements were amplified separately. Sometimes overlap extension PCR was used to combine them to larger constructs, but normally they were assembled by ligation assisted PCR. Classical restriction enzyme sites were added to the primers, the single PCR products were digested with the respective enzymes, ligated and the reaction products used as templates for subsequent PCRs.

For the constructs expressing the *ccdB* gene, *E. coli* strain DB3.1 was used, Mach1™-T1^R^ and SURE otherwise.

pGGE000 was converted into pGGH000 by cutting out the *Bsa*I recognition site and the E overhang via *Hind*III and *Bam*HI digestions and replacing it by an oligonucleotide pair providing the *Bsa*I site and the B overhang. pGGI000 was constructed similarly from pGGD000.

### Construction of Empty Destination Vectors

To create the GreenGate destination vectors, pGreen-IIS [14] was used as PCR template. The vector backbone was left unaltered, but the sequence between left border (LB) and right border (RB) was replaced by a short multiple cloning site. For the final constructs with the plant resistance cassette at the RB, the MCS had the orientation LB-*Xho*I…*Bam*HI-RB, and the orientation LB-*Bam*HI…*Xho*I-RB for the version with the plant resistance cassette at the LB. In the next step, the *Not*I and *Pae*I sites as well as the short spacer sequences in front of the borders were introduced via an oligo duplex (destroying the *Bam*HI/*Xho*I sites), again for both versions of the destination vector. The rest of the multiple cloning site was added in the third step, again using oligo duplices. Finally, the *caR-ccdB-lacZα* cassette flanked by two *Bsa*I sites in opposite orientation with the A and G overhangs was created via PCR and ligated into the *Kpn*I and *Xba*I sites of the pre-vector. For lack of transformants, only the final destination vector for the plant resistance cassette at the RB (pGGZ001) could be created that way, the one for the plant resistance cassette at the LB (pGGZ002) required an additional step. An intermediate *Bsa*I-*Xho*I-*Eco*RI-*Bsa*I oligo duplex was cloned into the *Kpn*I and *Xba*I sites of the prevectors and the PCR product then added via *Xho*I and *Eco*RI sites. Due to the observed instability of pGGZ002 in bacteria, we created pGGZ003 by PCR amplifying the *ccdB* cassette with exchanged *Xho*I and *Eco*RI sites thus inverting the *ccdB* cassette orientation but still retaining the plant resistance cassette at the LB design.

Bacterial host strains were DH5α (first vector), Mach1™-T1^R^ (all other intermediate vectors) and DB3.1 (final vectors).

To change the bacterial resistance in the destination vector from spectinomycin to gentamicin, the respective plasmids (pGGZ001, pGGZ002, pGGZ003) were amplified from the nucleotide directly downstream of the *spectinomycin adenyltransferase* (*specR*) gene stop codon to the nucleotide directly upstream of the start codon. External *Bsa*I sites were added to the primers. The *gentamicin acetyltransferase* (*gentR*) reading frame from *A. tumefaciens* strain GV3101 (pMP90RK) was amplified also with external *Bsa*I sites and compatible overhangs. Because this did not yield any transformants, a second PCR product was created additionally containing 69 nucleotides of the *gentR* 3′-UTR. *E. coli* cells transformed with the resulting plasmids (pGGY001– pGGY003) were grown in medium with 5 µg/mL gentamicin, for *A. tumefaciens* the respective concentrations ranged from 10 to 20 µg/mL.

Ampicillin and spectinomycin were used at 100 µg/mL, kanamycin at 50 µg/mL, chloramphenicol at 25 µg/mL and tetracycline (for pSOUP) at 5 µg/mL.

### Construction of the Intermediate Vectors for Two Constructs on One T-DNA

pGGM000 and pGGN000 were created from two PCR products each. pENTR1A was amplified without the attL sites and the *ccdB* cassette flanked by *Eco*RI and *Xho*I sites, the primers also providing the *Bsa*I recognition sites and the suitable overhangs. The *caR-ccdB-lacZα* cassette was also amplified with the same restriction sites at the end. The PCR products were digested with these enzymes and ligated.

### Methylated Oligonucleotide Duplex

The oligonucleotide duplex used in the more than two constructs on one T-DNA approach was created by annealing oligonucleotides A02827 (5′-taggaccttgagacCgaaaaggtggtctCa-3′) and A02828 (5′-atactgagacCaccttttcggtctCaaggt-3′). The nucleotides in capital letters were methylated.

### Creation of the GreenGate Entry Modules

In most cases the respective inserts were PCR amplified to create the GreenGate entry modules. The nucleotides 5′-AACA-GGTCTC-A-NNNN (nn)-3′ were added to the forward primer in front of the gene specific sequence. GGTCTC is the *Bsa*I recognition site, AACA was added because the enzyme does not cut if the restriction site is at the extreme ends of PCR products. NNNN represents the module specific overhang and 2 nucleotides (nn) are needed in case of the coding sequence and C-tag modules to bring the modules into frame (NNN represents an in-frame coding triplet in the overhangs). The sequence 5′-AACA-GGTCTC-A-NNNN-3′ was added to the reverse primers, followed by the reverse complement of the sequence of interest. NNNN stands for the reverse complement of the module specific overhang, the coding triplet being underlined.

After amplification, the PCR reactions were separated on agarose gels, the product bands excised, purified with innuPREP DOUBLEpure Kit (Analytik Jena AG, Jena, Germany) and digested with *Bsa*I. The respective empty entry modules (∼ 100 ng) were also cut with this enzyme, usually in the same tube (1 h, 37°C). The digestion was purified with the above mentioned kit and ligated with T4 DNA ligase (1 h room temperature, overnight 4°C). After heat-inactivation (10 min, 70°C) the reaction was transformed via heat shock into *ccdB* sensitive *E. coli* strains (Mach1™-T1^R^, DH5α, XL1-Blue MR). Transformants were checked by colony PCR, plasmid DNA was isolated from positive clones and checked by sequencing and test digestion.

If internal *Bsa*I recognition sites were present in the module sequence, they were removed by nucleotide substitution. For protein-coding sequences, silent mutations were chosen. In promoter and terminator sequences, the nucleotides to be changed were selected at random, but for later constructs we switched to always replace the first guanine by a cytosine. For simplicity, we used scar-free *Bsa*I-cloning to create the substitutions. Primers were designed on both sides of the internal *Bsa*I recognition sites introducing the mismatch and flanked on their 5′-ends by external *Bsa*I recognition sites. The overhangs generated by the external *Bsa*I cut were designed to be part of the gene-specific sequence and being different from the module specific overhangs.

Shorter modules were assembled as oligonucleotide duplices created from overlapping primers with unpaired 5′-overhangs complementary to the module specific overhangs. The oligonucleotides (10 µM or 100 µM) were mixed in equimolar ratios with each other, soused with boiling water, allowed to cool slowly down to room temperature and then ligated into *Bsa*I digested and purified entry vector.

### GreenGate Reaction

Plasmids were isolated from the bacterial hosts using the innuPREP Plasmid Mini Kit (Analytik Jena AG, Jena, Germany) according to the recommendations of the manufacturer. DNA was eluted with 100–125 µL elution buffer. The DNA concentration was usually not determined exactly but after gel electrophoresis estimated to be around 100 ng/µL judging from earlier preparations done with the same kit.

For the GreenGate reaction itself 1.5 µL plasmid of each of the six modules were mixed with 1 µL of the destination vector, 1.5 µL CutSmart Buffer (alternatively: FastDigest buffer), 1.5 µL ATP (10 mM), 1 µL T4 DNA ligase (30 u/µL) and 1 µL *Bsa*I-HF (alternatively FastDigest *Eco*31I) in a total volume of 15 µL. *Bsa*I-HF and CutSmart buffer were purchased from New England Biolabs GmbH, Frankfurt am Main, Germany, FastDigest *Eco*31I, FastDigest buffer, T4 DNA ligase and ATP from Fisher Scientific - Germany GmbH, Schwerte, Germany. Initially, we performed 50 cycles of 37°C for 5 minutes and 16°C for 5 minutes each, followed by 50°C for 5 minutes and 80°C for 5 minutes. Later we reduced the incubation time to 2 minutes each and the cycle number to 30. 6 µL of the reaction were used for heat-shock transformation of *ccdB*-sensitive *E. coli* (strains Mach1™-T1^R^, DH5α or XL1-Blue MR). 25–100 µL of competent cells (1.0–4.0*10^8^ cfu/1 µg pUC19 DNA/100 µL) were used and usually hundreds of transformants were recovered after plating (on two separate plates - 1/15 and the rest).

The products of the first GreenGate reactions were analyzed by both restriction endonuclease test digestions and sequencing of the ligation sites, later on by test digestions only.

When creating the intermediate supermodules for the two constructs on one T-DNA approach, ligase and ATP were added again after the GreenGate reaction and the mixture incubated for one more hour at room temperature before heat-inactivation and heat-shock transformation.

When using the methylated oligonucleotide pair, *Bsa*I was added after the GreenGate reaction and digestions carried out for one hour at 37°C prior to heat-inactivation and transformation.

### Troubleshooting

Usually the GreenGate system works quite reliably. In instances where the total number of colonies and/or the ratio of correct clones dropped, we could mostly attribute it to either bad enzyme quality or low DNA concentration of the modules and destination vectors. Since the activity of the restriction endonucleases suffers substantially from prolonged exposure to room temperature, we recommend aliquoting all enzymes and buffers to avoid repeated freeze-thaw cycles. Plasmid DNA minipreparations should be column purified, plasmid integrity checked by gel electrophoresis and their DNA concentration measured (100 ng/µl minimum). In case of frequently used elements, such as the destination vector or standard tags, we found midi preparations to be preferable and overall more reliable.

In case final constructs are obtained in which modules are ligated in arbitrary order, which suggests that 5′-overhangs were lost during the reaction, we suggest using a fresh enzyme batch. For some enzyme batches we found a reduction of enzyme amount by 50% to be very beneficial. Alternatively, using the long reaction program (50 cycles of 5 min at 37°C and 16°C each) helped to increase efficiency. When restriction enzyme activity is insufficient, plasmids with an integration of a complete entry vector with intact *Bsa*I sites might be recovered. This can be remedied by incubating with fresh *Bsa*I after the GreenGate reaction.

### *Nicotiana Benthamiana* Infiltration

*A. tumefaciens* strain ASE (pSOUP^+^) was transformed with the respective constructs. Transgenic clones were cultured in liquid selective LB medium for 2 nights at 28°C, resuspended in infiltration medium (10 mM MgCl_2_, 10 mM MES, 150 µM acetosyringone, pH 5.7) and pressed with a blunt syringe through the stomata at the abaxial site of leaves from approximately four weeks old plants [32]. The plants were put back to the incubators for 48 or 72 hours before analyzing the fluorescence levels.

For ethanol induction, plants were watered with a 1% v/v solution of ethanol.

Confocal laser scanning microscopy was done on a Nikon A1 Confocal Microscope with a 25× apochromatic lens, epifluorescence microscopy at a Zeiss Axio Imager.M1. Fluorescence intensities were measured with the ImageJ software.

### *Arabidopsis thaliana* Transformation and Plant Selection

*A. thaliana* plants were transformed by a modified version of the floral dip protocol [33], where only the tips of the inflorescences were dipped into the agrobacteria solution.

For selection on soil, Basta™ (glufosinate-ammonium; Bayer CropScience Deutschland GmbH, Langenfeld, Germany) was used at a concentration of 20 mg/L, both for spraying and watering. The concentrations of sulfadiazine, D-alanine, Basta™, kanamycin and hygromycin for selection on ½ MS plates were 0.75–7.5 µg/mL, 12 mM, 10 µg/mL, 50 µg/ml and 25 µg/mL, respectively.

### Plasmid Availability

All plasmids listed in Table 1 are available from Addgene (Massachussetts, USA) at www.addgene.org/cloning/greengate/lohmann. The sequence information has been deposited in GenBank under accession numbers KF718964 - KF719019.
